# New data for action. Data collection for KiGGS Wave 2 has been completed

**DOI:** 10.17886/RKI-GBE-2017-105

**Published:** 2017-09-27

**Authors:** Elvira Mauz, Antje Gößwald, Panagiotis Kamtsiuris, Robert Hoffmann, Michael Lange, Ursula von Schenck, Jennifer Allen, Hans Butschalowsky, Laura Frank, Heike Hölling, Robin Houben, Laura Krause, Ronny Kuhnert, Cornelia Lange, Stephan Müters, Hannelore Neuhauser, Christina Poethko-Müller, Almut Richter, Angelika Schaffrath Rosario, Jörg Schaarschmidt, Robert Schlack, Martin Schlaud, Patrick Schmich, Gina Schöne, Matthias Wetzstein, Thomas Ziese, Bärbel-Maria Kurth

**Affiliations:** Robert Koch Institute, Department of Epidemiology and Health Monitoring, Berlin

**Keywords:** CHILDREN AND ADOLESCENTS, GERMANY, HEALTH MONITORING, KIGGS, COHORT

## Abstract

The fieldwork of the second follow-up to the German Health Interview and Examination Survey for Children and Adolescents (KiGGS) was completed in August 2017. KiGGS is part of the Robert Koch Institute’s Federal Health Monitoring. The study consists of the KiGGS cross-sectional component (a nationally representative, periodic cross-sectional survey of children and adolescents aged between 0 and 17) and the KiGGS cohort (the follow-up into adulthood of participants who took part in the KiGGS baseline study). KiGGS collects data on health status, health-related behaviour, psychosocial risk and protective factors, health care and the living conditions of children and adolescents in Germany.

The first interview and examination survey (the KiGGS baseline study; undertaken between 2003 and 2006; n=17,641; age range: 0-17) was carried out in a total of 167 sample points in Germany. Physical examinations, laboratory analyses of blood and urine samples and various physical tests were conducted with the participants and, in addition, all parents and participants aged 11 or above were interviewed.

The first follow-up was conducted via telephone-based interviews (KiGGS Wave 1 2009-2012; n=11,992; age range: 6-24) and an additional sample was included (n=4,455; age range: 0-6). KiGGS Wave 2 (2014-2017) was conducted as an interview and examination survey and consisted of a new, nationwide, representative cross-sectional sample of 0- to 17-year-old children and adolescents in Germany, and the second KiGGS cohort follow-up.

The completion of the cross-sectional component of KiGGS Wave 2 means that the health of children and adolescents in Germany can now be assessed using representative data gained from three study waves. Trends can therefore be analysed over a period stretching to over ten years now.

As the data collected from participants of the KiGGS cohort can be individually linked across the various surveys, in-depth analyses can be conducted for a period ranging from childhood to young adulthood and developmental processes associated with physical and mental health and the associated risk and protective factors can be explored. As such, KiGGS Wave 2 expands the resources available to health reporting, as well as policy planning and research, with regard to assessing the health of children and adolescents in Germany.

## 1. Background and objective

**KiGGS Wave 2**

Second follow-up to the German Health Interview and Examination Survey for Children and Adolescents

**Data owner:** Robert Koch Institute

**Aim:** Providing reliable information on health status, health-related behaviour, living conditions, protective and risk factors, and health care among children, adolescents and young adults living in Germany, with the possibility of trend and longitudinal analyses.

**Study design**: Combined cross-sectional and cohort study conducted as an examination and interview survey

**KiGGS cross-sectional study**

**Population:** Children and adolescents with permanent residence in Germany

**Sampling:** Samples from official residency registries - randomly selected children and adolescents from the 167 cities and municipalities covered by the KiGGS baseline study

**Age range:** 0-17 years

**Sample size:** Approximately 15,000 participants

**KiGGS cohort study**

**Sampling:** Re-invitation of everyone who took part in the KiGGS baseline study (2003-2006; aged between 0 and 17 at that time) and who was willing to participate in a follow-up

**Age range:** 10-29 years

**Sample size:** Approximately 10,000 follow-up participants

**Survey period:** September 2014-August 2017

**Modules:** BELLA, EsKiMo, GerES, KiESEL, MoMo

More information is available at www.kiggs-studie.de/english

Health monitoring was established at the Robert Koch Institute (RKI) in 2008 [1, 2] to continuously monitor the health of the population living in Germany from birth to old age. Data is regularly collected for various age groups on physical and mental health, psychosocial risk and protective factors, health-related behaviour and health care as well as on health-related living conditions. The German Health Interview and Examination Survey for Children and Adolescents (KiGGS) is the key source of data for assessing the health of the next generation [3, 4].

KiGGS involves the regular completion of cross-sectional surveys that are representative for the children and adolescents living in Germany. At the same time, the participants of the KiGGS baseline study are also observed into adulthood as part of the KiGGS cohort. The regular collection of representative cross-sectional data enables estimates to be made of frequency (prevalence) rates for indicators of the health status of children and adolescents living in Germany who are aged between 0 and 17. The KiGGS study also provides the opportunity to analyse trends over time and links between various aspects, such as risk factors and medical conditions. Furthermore, data from the KiGGS cohort can be individually linked and therefore used to map health developments over the course of a person’s life and to analyse possible influencing factors. The transitional phases that occur within a person’s younger life – such as between childhood and adolescence and between adolescence and young adulthood – are particularly interesting in this regard, as are the possible causes and conditions that lead to changes in health.

The KiGGS data reflect differences in the health status and health-related behaviour of groups of children and adolescents. Particular focus is placed on vulnerable groups in order to ensure that the results can be used to improve health equity [5]. Moreover, the findings are integrated into policy advice provided within the Federal Health Reporting, are published in scientific publications, and are made available to researchers as public use files, which can be requested at the Robert Koch Institute for scientific and non-commercial use.

The first KiGGS study was carried out between 2003 and 2006 as a combined examination and interview survey (the KiGGS baseline study) as a consequence of the previously scarce and heterogeneous data on the health of children and adolescents in Germany. It resulted in data being collected from 17,641 children and adolescents aged between 0 and 17 at 167 sample points in Germany (participation rate: 66.6%) [6]. In a new approach, children from families with a migration background were included. In order to do so, a number of different measures were taken. The aim was to reflect the migrants’ distribution within the German population [7, 8]. KiGGS was initiated as a cross-sectional study and its breadth and depth remain unique in Germany. As a result of this study, many questions about the health of children and adolescents were able to be answered for the first time and new hypotheses could be developed. The first telephone-based follow-up survey (KiGGS Wave 1) was undertaken between 2009 and 2012 with a reduced and partly modified range of indicators on health status and health-related behaviour [9, 10]. KiGGS Wave 1 combined a cross-sectional and longitudinal design [9]. Girls and boys who had taken part in the baseline survey and who had stated that they were willing to take part in a follow-up (they were now aged between 6 and 24) were invited to participate again (first follow-up of the KiGGS cohort: n=11,992; participation rate: 68.5%). In addition, follow-up participants aged between 7 and 17 were also incorporated into the cross-sectional sample (n=7,913; participation rate: 72.9%). Furthermore, a new sample covering 0-to 6-year-olds, which was drawn from official population registers from the KiGGS baseline sample points (n=4,455; participation rate: 38.8%), supplemented this population. In order to be able to provide representative conclusions from this combined cross-sectional sample, weighting factors were employed to account for the varying readiness to participate in the study and to ensure that the data reflect the current structure of the population in Germany [9].

Data collection for the second follow-up survey (KiGGS Wave 2) was carried out between September 2014 and August 2017. This article describes the study design, sample, survey methods as well as the study’s content and the opportunities opened up by KiGGS Wave 2 for further analysis.

## 2. Methodology

### 2.1 Study design and sampling

**Figure 1:**
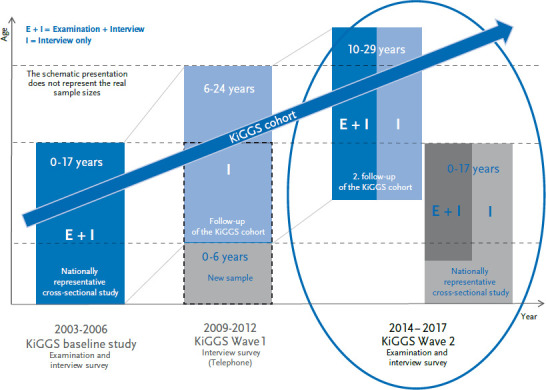
The design of the KiGGS study Own diagram

Two studies were combined within the framework provided by KiGGS Wave 2: 1) a nationwide representative cross-sectional study of 0 to 17-year-old children and adolescents living in Germany; and, 2) the second follow-up of the KiGGS cohort. In line with the KiGGS baseline study, KiGGS Wave 2 was carried out as a combined examination and interview survey. Whereas questionnaires were sent to all participants, examinations were only carried out for a subpopulation (Figure 1).

In order to be able to provide up-to-date prevalence estimates among children and adolescents aged between 0 and 17 with a primary residency in Germany, a new sample, stratified for age was requested [11]. It was sourced from addresses held by the official population registers located in the 167 sample points of the baseline study. A randomly allocated subsample of 3-to 17-year-olds was invited to an examination and interview, a further subsample of 0-to 17-year-olds was invited to fill in a questionnaire only.

All participants of the KiGGS baseline study were invited to take part in the follow-up of the KiGGS cohort, irrespective of whether they had participated in KiGGS Wave 1. However, only participants who had stated that they were willing to be interviewed again, who could be located, and still lived in the original sample point were invited to an examination and interview for KiGGS Wave 2. Potential follow-up participants who had moved away from the original sample point were invited to an interview only. People who did not wish to or could not participate in the examination were asked to fill in the questionnaire. Participants aged 18 or above who had not yet participated by mid-May 2017, were contacted again and given the opportunity to take part in an online interview.

### 2.2 Assessment methods and testing instruments

**Figure 2:**
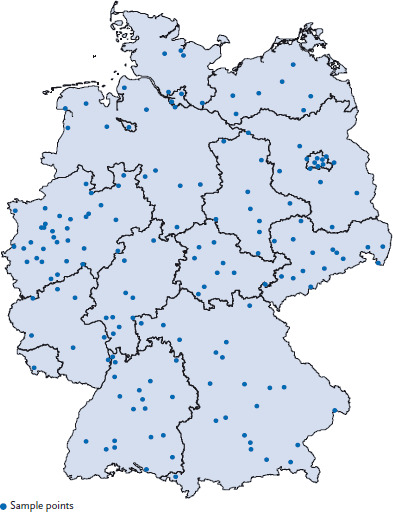
Sample points of KiGGS Wave 2 Source: RKI

In order to conduct examinations for the study, three study teams working in parallel visited 167 sample points (Figure 2). The teams constructed temporary examination centres in rented rooms. In the temporary examination centre, the teams brought and built up their equipment. Each team consisted of a physician, two examiners and an assistant at the reception. The team started by providing information to parents and participants about the study’s context and content, data protection and the individual scope of the examination. All examinations were carried out by trained staff after written informed consent had been provided and as long as there were no reasons for exclusion or contraindications.

The examination routine varied depending on age and whether the participant belonged to the cohort or the cross-sectional study (Table 1). Various examinations and tests were carried out, which are described more detailed later in this article. A computer-assisted medical interview was used to ask questions about physician-diagnosed conditions. An interview was also conducted on the use of medication to gather data on the (prescribed) medicines and food supplements that had been taken over the last 7 days preceding the interview. The participants brought their medication with them and the central pharmaceutical number on the packaging was scanned and recorded using the AmEDa drug identification database [12]. The vaccination records, which the participants also brought with them, were copied so that the information they contained could be entered into a database at a later date. Blood and urine samples were collected and analysed and serum and urine samples were stored for analysis at a later time only if further informed consent had been provided to do so. An overview of the laboratory parameters that were analysed can be found in Table 2.

KiGGS Wave 2 employed written, paper-based, age-adjusted questionnaires on health and nutrition. These were provided to the parents, children, adolescents and young adults (Table 1). The parents of participants aged between 0 and 17 were asked to complete an age-adjusted questionnaire on health, and a questionnaire on nutrition for participants aged between 3 and 10 years of age. From the age of 11, the children, adolescents or young adults themselves filled out age-specific variants of a health questionnaire and a questionnaire on nutrition. A small number of cohort participants were 10 years old during the invitation process. These participants were treated in the same manner as 11 year olds. The content of the medical interview was integrated into the questionnaire on health for those who did not participate in the examination. The young adults belonging to the KiGGS cohort were also offered an online variant of the health questionnaire at a later date, as described earlier in this section (see 2.1).

### 2.3 Participant recruitment

Participant recruitment for KiGGS Wave 2 followed a plan drawn up at the beginning of the study. The 167 sample points were visited systematically to avoid combining seasonal and regional effects as far as possible [13]. Three sample points were visited at the same time. The participants were divided into four subgroups (‘cross-sectional examination and interview’, ‘cross-sectional interview’, ‘cohort examination and interview’ and ‘cohort interview’) and the invitations were normally sent out six weeks before examination (Figure 2). Whereas invitations to minors were addressed to parents or guardians, potential participants aged 18 or above received the invitation directly. People who did not respond to the invitation received a reminder approximately two weeks after the initial invitation letter. If this did not produce a response, the reminder was followed up by a phone call or home visit.

Parents and adult participants who were only invited to an interview received the questionnaires on paper gether with their invitation, information brochure and consent form. They were asked to return the completed questionnaires to the RKI within two weeks. In the case of children and adolescents aged 11 or above, the questionnaires were not sent out until the parents had provided written consent for an interview to be conducted with a minor. The people invited to the examination received their questionnaires together with a confirmation of their appointment as soon as a date had been agreed upon by telephone. The participants were asked to bring their completed questionnaires to the examination centres. Parents and young adults who had declined to participate in the study received a short non-responder questionnaire so that basic information about socio-demographic and health-related characteristics could be gathered. These data were used to compare participants with non-participants in order to identify any systematic differences between responders and non-responders.

After having received detailed information about the study, all potential participants were asked to provide their written informed consent; they either did so in the examination centre or, in the case of participants who were only surveyed via questionnaire, by post. Minors’ consent forms were signed by a parent or guardian; adolescents aged 14 or above signed their own forms but had them countersigned by a parent or guardian; participants aged 18 or above signed the informed consent themselves. As an expression of appreciation, the participants were provided with an age-appropriate incentive. Everyone who underwent an examination also received a written medical report six to eight weeks later detailing their test and laboratory results.

In line with the KiGGS baseline study, measures were taken to integrate children and adolescents with a migration background into the new cross-sectional sample in numbers that reflected the structure of the German population; measures were also put in place to encourage KiGGS cohort participants with migration background to further take part in the study [8]. This included migrant-specific public relations in the sample points, improving intercultural competence among staff with direct contact to the participants, and – for potential participants invited for the first time – the offer of having their invitation material, and, if applicable, the parents’ questionnaires, provided in English, Turkish, Russian or Serbo-Croatian. A higher proportion of quality-neutral sample loss can be expected among children and adolescents without German nationality (as invitations are more likely to be returned marked with ‘addressee no longer at given address’ or ‘address unknown’) [14]. Therefore, participants with a nationality other than German were oversampled for the new cross-sectional sample by a factor of 1.5.

### 2.4 Quality assurance

In order to guarantee a high level of quality during the data collection phase of KiGGS Wave 2, a multi-stage quality assurance system for fieldwork, laboratory analysis, and data entry, storage and processing was implemented. Detailed manuals stipulated the standard operating procedures (SOP) and the quality assurance measures that were to be put in place at each step of the process in all of these areas.

All actors involved in the study attended a comprehensive training programme explaining the guidelines set out in the manuals before starting fieldwork. Follow-up training was regularly carried out during the data collection phase as required. The examination and interview component, which was tailored to age range and cross-sectional or cohort participation, was carried out in an SOP-compliant, standardised manner. By participating in ring trials and other pre-determined quality control measurements, which were undertaken before the actual analyses took place. It was possible to ensure a high quality laboratory testing by the RKI’s accredited Central Epidemiological Laboratory. In order to avoid errors during data input, the highest possible level of standardisation was carried out both when measurements and test results were gained in the study centres as well as during data entry from the written questionnaires at the RKI. Among other factors, automated checks were performed to identify incorrect, contradictory, and incomplete data. The questionnaires were produced as machine-readable forms that could be scanned, validated and exported to a database at the RKI. Data quality was continuously monitored by the RKI’s Epidemiological Data Centre and Research Data Centre. This included entering the data from a proportion of the questionnaires twice. In addition, a large number of further quality assurance measures are to be carried out in the context of data processing before the data is to be made available for scientific evaluation.

Finally, the implementation of the study was regularly accompanied by internal and external quality assurance mechanisms. The evaluations and recommendations for action that this led to contributed to the optimisation of processes, and further training for staff.

### 2.5 Weighting

A weighting factor was created to ensure that prevalence estimates from the cross-sectional component of KiGGS Wave 2 are nationally representative in terms of age and gender distribution within the federal states, as well as with regard to parental levels of education and nationality (‘German: Yes/No’). This weighting also accounts for the different probabilities of participants taking part and corrects for deviations of the design-weighted net sample from the German population using demographic statistics from 2014/2015 and levels of educational attainment in accordance with the CASMIN (Comparative Analysis of Social Mobility in Industrial Nations) system of classification [15] obtained from the 2013 microcensus.

For the longitudinal component of KiGGS Wave 2 (the KiGGS cohort), the weighting procedure also accounts for differences in the study participants’ likelihood to take part in the follow-up. As was the case with KiGGS Wave 1 [9], the probability that a participant would take part in a follow-up is estimated using a weighted logistic regression model with the probability of participating in a follow-up as the target variable alongside other variables from the baseline study (socio-demographic characteristics, health status and health-related behaviour) as explanatory variables. This reduces the level of bias in the study population caused by selective non-participation, at least as long as non-participation can be predicted by the data on socio-demographic and health-related indicators gathered for the KiGGS baseline study.

### 2.6 The modular character of KiGGS Wave 2

**Figure 3:**
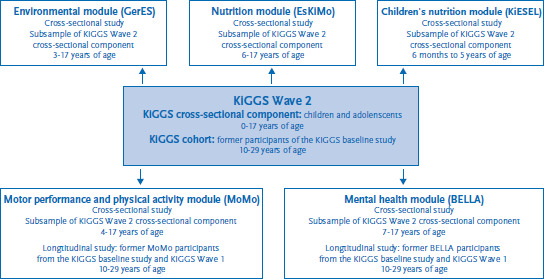
Modular structure of KiGGS Wave 2 Own diagram

The core survey of KiGGS Wave 2 described here is supplemented by five independent modules conducted as subsamples of the core survey (Figure 3). Each module consists of an in-depth study focused on a particular theme; the data gathered for the modules can be linked to the data from the KiGGS cross-sectional component or the KiGGS cohort (see the articles on the BELLA, EsKiMo, GerES, KiESEL and MoMo modules elsewhere in this issue).

### 2.7 Data protection and ethics

All of the RKI surveys strictly observe the data protection regulations set out in the German Federal Data Protection Act. The Hanover Medical School’s ethics committee assessed the ethical questions raised by KiGGS Wave 2 and granted it ethical approval (No. 2275-2014). The Federal Commissioner for Data Protection and Freedom of Information also had no reservations about the study. Participation in the study was voluntary. The participants and/or their parents or guardians were informed about the study’s aims and content, as well as data protection, and provided their informed consent.

## 3. Content and instruments of the survey

In KiGGS Wave 2, data on a wide spectrum of health-relevant topics that are related to specific phases of life were collected from birth to childhood and from adolescence to young adulthood (Tables 3-7). The instruments and methods that were applied were kept as constant as possible during the KiGGS waves, both in terms of the identification of population-related trends in the health situation of children and adolescents in Germany, as well as to allow analyses of health-related developments over the course of a person’s life within the framework of the KiGGS cohort. KiGGS Wave 2 expanded the study’s focus to adequately reflect recent developments relevant to public health and to enable more differentiated analyses to be conducted. This includes the addition of an in-depth module as part of the KiGGS cohort aimed at identifying family-related and health care factors associated with the development, progression and impact of psychological disorders (especially ADHD), obesity and allergic diseases (especially asthma).

### 3.1 Physical health

With regard to physical health the aim of the study is to assess the development of individual diseases with a high level of public health relevance, chronic disease in general, and trends in physical risk factors among children and adolescents in Germany in a lifecourse perspective (for the content of the survey and the instruments it employs, see Table 1, Table 2, Table 3).

The prevalence, time trends and the consequences of overweight and obesity are important parameters in child and adolescent health. As such, everyone who participated in an examination had their body height, body weight and waist circumference measured. In order to provide a detailed analysis of obesity, these measurements gathered during KiGGS Wave 2 were supplemented by bioimpedance measurements to determine body composition (such as body fat percentage) in adolescents and young adults. As part of the in-depth KiGGS cohort module on life-course developments of chronic conditions such as obesity, data was gathered retrospectively on treatments that participants had undergone due to physician-diagnosed obesity.

The measurements set out above were supplemented by information on the period ranging from birth to adolescence. Unless this information was already known (as was the case with cohort participants), the gestational age, birth weight and size as well as parameters of pubertal maturation were recorded. A language screening test was introduced in KiGGS Wave 2 that allows an evaluation of the language development of children aged between 3 and 5 for the first time.

As a potentially modifiable cardiovascular risk factor in childhood and adolescence, resting blood pressure and heart measure were measured in a standardised manner in line with the measurements of the baseline study. In order to identify subclinical changes in the arterial walls (subclinical arteriosclerosis), carotid intima media thickness (CIMT) was measured for the first time using an ultrasound device. This was performed on cohort participants aged 14 and older. Furthermore, urine and blood samples were examined for known risk factors of cardiovascular diseases (such as disorders of lipid and glucose metabolism).

The standardised computer-assisted medical interview focused on selected physician-diagnosed diseases, time of diagnosis and treatments. Participants who did not take part in the examination part answered the questions via a questionnaire. Additional disease-specific questions were asked in the questionnaires on health; information on the medical treatment received in these cases can be gathered from the interviews on medication.

KiGGS also focuses on allergies and asthma, as well as the course of these conditions throughout childhood and adolescence. The survey method employed to obtain the data in these cases – information provided by the participants, and laboratory tests of their sensitivity to selected allergens – has remained unchanged across age groups and survey periods. Therefore time trends can be analysed. The lifecourse development of atopic diseases is also the subject of an in-depth module undertaken as part of the KiGGS cohort. KiGGS Wave 2, therefore, also covers not only factors associated with asthma but also self-management of asthma, asthma control and treatment aspects.

Urine and blood samples were analysed for thyroid hormones, vitamin D and other laboratory parameters. Further topics of the written questionnaire were self-reported chronic conditions and associated functional limitations, as well as family predispositions to allergies or cardiovascular disease. Other issues included chronic pain, accidents requiring treatment, and – for adult cohort participants – sexual health. Adult cohort participants were also interviewed about any serious illnesses or accidents that they or their parents had experienced.

### 3.2 Mental health

Since the spectrum of diseases affecting children and adolescents has shifted in the last few decades towards chronic diseases and functional and mental disorders [16], it is essential that population-based epidemiological studies also monitor the mental health of this age group. The concept developed for the assessment of mental health in childhood and adolescence and its persistence into adulthood includes gathering data on subjective assessments of Health Related Quality of Life (HRQoL), psychological disorders and symptoms of mental disorders. In addition, data was also collected on self-reported and parent-reported medical or psychotherapeutic diagnoses, as well as associated risk and protective factors and aspects of health care. This data was gathered using written questionnaires based on tested, standardised instruments (Table 4).

In order to estimate current prevalence rates and to provide up-to-date information on time trends among psychopathological problems and mental disorders (e.g. attention deficit/hyperactivity disorder ADHD or eating disorders) as well as the subjective health of children and adolescents in Germany, identical instruments were used across all survey waves. The way in which psychosocial protective factors, such as self-efficacy, personal resources, family environment and social support, were surveyed as factors that positively influence health and well-being also remained largely unchanged. Trend analyses of HRQoL, in contrast, are only possible between KiGGS Wave 1 and KiGGS Wave 2, since the baseline study employed a different survey instrument.

Since KiGGS Wave 1, screening instruments and medical and psychotherapeutic diagnoses have been used to gather data from adults participating in the KiGGS cohort on symptoms of depression and anxiety disorders. In addition, data was also collected for an in-depth KiGGS Wave 2 module on the level of health care that participants had received up to this point of time for three selected mental disorders: ADHD, depression, and anxiety disorders. These information was gathered retrospectively. In addition, the survey concept drawn up for the assessment of the mental health of participants belonging to the KiGGS cohort was expanded to include questionnaires on personality issues and a person’s level of satisfaction with their life. Finally, data was also gathered on mental disorders affecting the participants’ biological parents, participants’ experiences of discrimination and adverse or traumatic childhood experiences.

### 3.3 Health-related behaviour

Healthy behaviours are frequently established during childhood and adolescence and extend into adulthood. Therefore, the KiGGS study identifies different areas of health-related behaviour using test procedures and detailed survey elements (Table 1, Table 5). It also collects information on diet, physical and sporting activity as well as the use of certain substances (such as tobacco or alcohol).

The nutritional behaviour of children, adolescents and young adults was assessed as part of the core study of KiGGS Wave 2 in line with the baseline study via the Food Frequency Questionnaire (FFQ) [17]. The data gathered via the FFQ enables key indicators of nutritionrelated behaviour to be mapped, including fruit and vegetable consumption, and the consumption of sugar-sweetened beverages. It also means that nutritional indices can be created such as on healthy nutrition among children and adolescents [18]. A more in-depth study of nutritional behaviour is carried out as part of the two modules on nutrition: KiESEL for children between 6 months and 5 years of age, and EsKiMo for children and young people between 6 and 17 years of age. Data was also gathered on breastfeeding and early childhood nutrition within the health questionnaire. As such, trend analyses can be carried out on the proportion of breastfed children and on the duration of breastfeeding in Germany. In addition, the longitudinal design of the KiGGS cohort can be used to investigate whether breastfeeding influences future health.

Physical activity behaviour was assessed using self-reported information on sports and exercise performed during leisure time and in sports clubs as well as on daily physical activity levels (compliance with the physical activity recommendations of the World Health Organization, WHO) and active transportation. In addition, physical activity was objectively measured among participants taking part in the longitudinal component using an accelerometer. In order to assess motor skill performance, motoric tests (such as standing on one leg) were carried out with 4-to 10 year-old children and cardiorespiratory fitness was measured using a submaximal cycle ergometer test for children and adolescents aged 11 or above. Developments over time and trends in sporting activity can be analysed over two or three KiGGS study points, as can compliance with the WHO recommendations, and levels of motor skill performance and fitness. With respect to substance use, temporal trends and individual trend analyses are possible for smoking among young people and young adults as comparable instruments were used over three survey dates. As of KiGGS Wave 1 questions about the frequency and number of cigarettes smoked were asked as well as questions about the use of water pipes (shisha pipes), and e-cigarettes. In addition, all three KiGGS surveys included questions about the participants’ exposure to passive smoking. In the case of alcohol consumption, temporal developments and trends in lifetime prevalence can be examined over the three KiGGS survey waves. Trend and progression analyses can be carried out with harmful levels of alcohol consumption and binge drinking, using data from KiGGS Wave 1 and Wave 2. Furthermore, the participants’ parents were questioned about their smoking habits and levels of physical activity.

### 3.4 Health care and prevention

As data was collected in the same manner during the various KiGGS waves, it can be used to study temporal trends in various aspects of health care, including the use of outpatient medical and therapeutic services, the use of inpatient health care facilities in the last twelve months, childhood and adolescent screening and dental check-ups.

The participants’ vaccination status – an important preventive measure against infectious diseases – was also recorded in line with the method used in the KiGGS baseline study. Once the vaccinations, as stated in the participants’ vaccination records, had been entered into a database, vaccination status was defined in accordance with recommendations made by the Standing Committee on Immunisation (STIKO). The parents’ questionnaire also asked questions about why vaccinations had not been carried out (if applicable). Serum samples were used to check for the presence of antibodies against vaccine-preventable diseases, such as measles, mumps, rubella, chickenpox and hepatitis A and B.

Information on survey content and the instruments can be taken from Table 6.

### 3.5 Social, familial and environmental determinants of health

Due to the importance of socio-economic status for the health-related opportunities of children and adolescents, it is particularly important to conduct a comprehensive survey of a family’s social situation [19]. Therefore, standardised questions were asked about parents’ income, education and profession in order to determine a family’s socio-economic status. This involved the use of a multidimensional index similar to that used for KiGGS Wave 1 [20]. In the case of adolescents, data were also collected on social factors such as the form of school they attend, the type of school-leaving certificate they have received or expect to receive and individual school achievements. Parents of children up to 10 years of age provided data on the duration and subjective assessment of the quality of care they receive outside of the family. Adolescents aged 11 or above also provided a subjective assessment of their family’s social status. As with the parents, young adults belonging to the KiGGS cohort answered questions about income, education and occupation; but they also provided a subjective assessment of their own social status. In addition, data was gathered retrospectively about the educational or occupational situation of this group for each year of a participant’s life from the age of 15.

In order to provide for a more differentiated form of data collection with regard to family background, the parents and participants (aged 11 or above) were asked to provide information about their current family form, household composition, (parental) marital status, the number and order of biological siblings. Additionally, the atmosphere within their family was assessed by parents and children (aged 11 or above). The survey concept developed to gather data on familial influencing factors was expanded for the KiGGS cohort by an in-depth study about life in patchwork families, parenting style as well as the personality, well-being and stress faced by the parents as further psychosocial components of the family environment. Questions were also posed about critical events in a person’s life, such as separation from or death of a parent and the exact dates that these events occurred. Finally, where applicable, data was gathered on when a participant had moved out of his or her parents’ home and about the participant’s partner.

The existence of a migration background was determined – as in previous surveys – using the data on the country in which the participant or his or her parents were born, and parental nationality [8, 21]. Data was also gathered on the year of immigration, residence status, the language spoken at home, and the specific group of migrants that the person belonged to. This enables a differentiated analysis to be conducted depending on a one- or two-sided migration background [8], the length of stay, the immigrant generation and a participants’ legal status.

With regard to environmental determinants of health, KiGGS Wave 2 sought the participants’ consent to link their current and previous addresses, including the length of time that they had lived in a specific location, to data about the condition of their (past) residential environments, such as particulate matter concentration, traffic noise, as well as how far they lived from health care facilities and green spaces. Questions were also asked about the home environment and the neighbourhood, such as the opportunities that exist for exercise, play and sport, but also about factors such as environmental pollution (e.g. noise).

An overview of survey content and the instruments which assess social, familial and environmental determinants of health are given in Table 7.

## 4. Discussion and outlook

The tasks of the RKI as a national public health institute include monitoring, protecting and improving the health of the population. The RKI uses a valid database to detect health problems among the population at an early stage, assess developments and problem areas, and identify approaches to health promotion and disease prevention in line with its mission statement ‘Recognition – Evaluation – Action’ [4]. The RKI uses a wide range of sources for this purpose, including data from health insurance companies, service providers, disease registers, reporting undertaken in accordance with the Protection against Infection Act, official statistics (such as cause-of-death statistics), and data from hospital statistics and regional epidemiological studies. However, there are still gaps in the data, such as on social influencing factors and their associations with health status, health-related behaviour and environmental influences. In particular, healthy people who do not require any of the above-mentioned systems of care provision constitute the remaining ‘unchartered waters’ on the health indicator atlas. The Health Monitoring system was established at the RKI to fill in these gaps – it is financed by the RKI and the Federal Ministry of Health (BMG) [1, 2, 22]. The regular implementation of population-representative health surveys in Germany that focus on the entire age range within the population in Germany enables comprehensive assessments to be made of health at the population level and of developing trends. Furthermore, the regularly conducted representative KiGGS cross-sectional surveys and the longitudinal component of the KiGGS cohort provide an indispensable source of data on children and adolescents.

Data was collected on a broad spectrum of health indicators for the KiGGS baseline study (2003-2006) using objective measurements, tests, laboratory analyses and supplementary representative surveys of children aged between 0 and 17 years living in Germany at that time. The data enabled the creation of numerous reference value tables, such as on laboratory parameters or anthropometric measurements and blood pressure levels [23, 24]. The results are highly relevant to public health. For the first time population-based prevalences of children’s accidents, allergies and bronchial asthma, mental disorders and attention deficit/hyper-activity disorder (ADHD) could be assessed [25-28]. The strong increase in levels of obesity among adolescents since the 1980s, the rise in mental disorders, insufficient levels of physical activity and an unhealthy diet among certain risk groups of children and adolescents are particularly worrying [5, 29]. In addition, the results showed the significant influence that a family’s social situation can have on children’s and adolescents’ health [19, 30-32].

The results of KiGGS Wave 1 (2009-2012) demonstrated that the prevalence of frequently occurring chronic health conditions in children and adolescents [33-37] identified during the baseline remained largely constant. However, the study also identified positive developments. For example, a higher proportion of children had visited a paediatrician at least once in the previous year, which can be explained by the introduction of new recommendations on vaccination and check-ups, as well as a higher rate of participation in screenings [38]. The reduced smoking rates among teenagers and of maternal smoking during pregnancy, as well as the lower levels of passive smoke exposure among children and adolescents during this period were also very welcome and are demonstrative of the success of increased policy efforts to curb smoking and to improve the protection of non-smokers [39-41].

The results of the KiGGS study provide important starting points for policy measures aimed at promoting child health and improving medical care. The findings of the KiGGS study led the RKI and the German Federal Center for Health Education (BZgA) to joint recommendations for health promotion and disease prevention. The resulting age-specific recommendations were addressed in various policies and programmes including health policies [5, 42].

The statutory provisions on examinations for the early detection of diseases (“U” screening) enshrined within the Act to Strengthen Health Promotion and Preventive Health Care (Preventive Health Care Act, PrävG), which came into force on 25 July 2015, provide a more recent example of the practical implementation of findings from the KiGGS study. The Preventative Health Care Act significantly expanded the scope for action in health promotion and disease prevention among children and adolescents in line with the challenges identified by KiGGS. The study also constitutes an important source of data for the evaluation of preventive measures. In addition to the structures defined in the Preventive Health Care Act and new institutions such as the National Prevention Conference and the Prevention Forum, it is essential to promote the sharing of experiences and networking between the numerous actors involved in the large range of existing relevant activities. In order to do so, the Federal Ministry of Health, together with the BZgA, organised the ‘Forum for Promoting Health and Prevention for Children and Adolescents’ in Berlin on 22 February 2017, and launched an exchange aimed at enabling experts to anchor prevention and health promotion among children and adolescents more strongly in their respective fields and to strengthen these issues at the practical level. In the long term, these activities are aimed at establishing a platform on child and adolescent health at the Federal Ministry of Health with the special focus on disease prevention and health promotion.

The KiGGS results were also of central importance during the review and expansion of the ‘Grow up healthy’ health goal: the study’s findings led to the incorporation of new aspects such as vaccination, accident prevention and mental health, and the goal now focuses more strongly on equity in health [43]. The health goal ‘All about birth’, which was adopted in 2017, is intended to contribute towards ensuring healthy development in later life by focusing on prenatal development and a child’s first year of life [44]. The KiGGS data will also play a major role in reviews of other health goals that are applicable to children and adolescents.

A lot of interest has already been expressed in the latest KiGGS results: Are developments heading in the right direction? Are the various efforts that are being undertaken in numerous areas of society to improve children’s health bearing fruit? What has become of the participants of the KiGGS cohort over the last eleven years?

The new representative cross-sectional component in KiGGS Wave 2 for children and adolescents aged between 0 and 17 currently living in Germany enables studies on trends in health status, health-related behaviour and the uptake of health care services to be updated. The follow-up of the people who participated in the KiGGS baseline study (KiGGS cohort) opens up the possibility of conducting genuine life-course research, as it constitutes the first population-wide cohort study in Germany that begins in childhood and adolescence. Assuming that the foundations for good health in old age are laid in childhood, the results should provide the foundation for healthy aging.

The first publication of the selected results from KiGGS Wave 2 is planned for March 2018. Evaluations of the KiGGS data are to be published in stages in the Journal of Health Monitoring as part of the RKI Federal Health Reporting. In addition to cross-sectional and trend analyses, longitudinal analyses will be presented. The June 2018 issue of the Journal will focus on health-related behaviour, with the September issue concentrating on the physical and mental health of children and adolescents. Both issues will include analyses of social inequality in the respective subject areas. Complementary and in-depth publications are also planned for other scientific journals. The interest shown in the new study is reflected in the fact that the RKI’s research data centre will be providing the data as a public use file. This should ensure that the results of KiGGS Wave 2 provide an important contribution to improving the health of children and adolescents in Germany.

## Key statements

- KiGGS is conducted within the Health Monitoring system at the Robert Koch Institute; it is the key source of nationwide data on the health of children and adolescents in Germany.
- KiGGS Wave 2 was conducted between 2014 and 2017 and is the second follow-up to have been carried out as an examination and interview survey in line with the initial KiGGS baseline study.
- KiGGS provides information on health status, health-related behaviour, psychosocial risk and protective factors, as well as health care utilisation.
- The nationwide representative cross-sectional component of the KiGGS study enables up-to-date prevalence estimates, correlation analyses and trend analyses.
- The longitudinal component – the KiGGS cohort – provides information on health-related developments while taking into account social and individual influencing factors.

## Figures and Tables

**Table 1:** Survey methods of KiGGS Wave 2

	Cross-sectional study			Cohort study	
	0-2 years	3-10 years	11-17 years	10-17 years	18-29 years
**Examination component**					
Measurement of height, weight and waist	No examination	X	X	X	X
Bioimpedance measurement			14-17	14-17	X
Ultrasound examination of carotid artery intima media thickness (CIMT)				14-17	X
Measurement of resting blood pressure and heart rate		X	X	X	X
Blood and urine samples		X	X	X	X
Language screening		3-5			
Motor function tests		4-10			
Cycle ergometer test (lactate measurement only age 14 and over)			X	X	X
Copy of vaccination record		X	X	X	X
				X	X
Interviews on medication (AmEDa, standardised recording of medicines and food supplements [12])		X	X	X	X
Standardised computer-assisted medical interview		X	X	X	X
**Interview component**					
Health questionnaire (version for parents)	X	X	X	X	
Health questionnaire for participants			X	X	X
Food frequency questionnaire (version for parents)		X			
Food frequency questionnaire for participants			X	X	X
Questionnaire on diseases (version for parents)		X^*^	X^*^	X^*^	
Questionnare on diseases for participants					X^*^

^*^ Only participants who did not take part in the examination component (instead of the computer-assisted medical interview)

**Table 2:** Laboratory parameters analysed in KiGGS Wave 2 (from blood, serum or plasma, unless stated otherwise)

	Cross-sectional study			Cohort study	
	0-2 years	3-10 years	11-17 years	10-17 years	18-29 years
Sodium, potassium, calcium, phosphate, alkaline phosphatase, total protein, creatinine, liver enzymes, ferritin, hs-CRP, 25(OH)D, TSH, fT3, fT4, cholesterol, LDL and HDL cholesterol, triglycerides, hepatitis B antibodies including anti-HBs and anti-HBc, HBs antigen, hepatitis A antibodies, herpes simplex type 1 antibodies	No examination	X	X	X	X
Antibodies to herpes simplex type 2			16-17	16-17	X
Antibodies to Helicobacter pylori		8-10	X	X	X
Antibodies to hMPV and para-influenza		X*	X*		
Allergic sensitisation to various allergens, total IgE		X	X	X	X
HbA_1c_				14-17	X
Antibodies to measles, mumps, chickenpox		X	X	X	X
Antibodies to rubella		X	X		
Antibodies to human respiratory syncytial virus and various respiratory pathogens		X			
Antibodies to influenza		X	X		
Antibodies to Borreliosis		X	X	X	X
Antibodies to Toxoplasma gondii		♀	♀	♀	♀
Glucose, iodine, sodium, potassium and creatinine in urine		X	X	X	X

♀ Only for female participants

* On a subsample

Hs-CRP=high-sensitivity C-reactive protein, 25(OH)D=25-hydroxy vitamin D, TSH=thyroid stimulating hormone, fT3=free triiodothyronine, fT4=free thyroxine, LDL cholesterol=low density lipoprotein cholesterol, HDL cholesterol=high density lipoprotein cholesterol, anti-HBs=antibodies to the hepatitis B-surface antigen, anti-HBc=antibodies to the hepatitis C-antigen, HBs antigen=hepatitis B surface antigen, hMPV=human metapneumovirus, Total IgE=immunoglobulin E, HbA1c=glycated haemoglobin

**Table 3:** Contents of interviews and instruments of KiGGS Wave 2 to survey physical health

	Cross-sectional study			Cohort study	
	0-2 years	3-10 years	11-17 years	10-17 years	18-29 years
**Physical health**					
Participant provided information on height and weight^A^; Perception of own body^B^; Weight and height of parents^C^; Weight and height of partner^D^; Treatment received for diagnosed obesity^E^	A C	A B C	A B C	A B C E	A B D E
Size and weight at birth, gestational age^A^; Birth by caesarean section^B^; Parameters of pubertal maturation^C^	A B	A B C (from 7)	A B C	B C	B
Subjective health (Minimal European Health Module, MEHM [45])^A^; Self-reported chronic disease (MEHM [45])^B^; Functional limitations caused by health problems (item from: Children with Special Health Care Needs (CSHCN) Screener [46])^C^; Disability^D^; Incapacity to work^E^; Visual and hearing impairment^F^	A B C D F	A B C D F	A B C D F	A B C D	A B C D E
Questions concerning selected physician-diagnosed diseases/risk factors and their treatment (included in the medical interview or the questionnaire on diseases): hay fever, neurodermatitis, asthma, allergic contact dermatitis, heart disease, diabetes, epilepsy, obstructive bronchitis, migraine^A^; Disorders of lipid metabolism, hypertension, cancer^B^; Congenital malformations^C^	A C	A C	A C	A B	A B
Asthma control (asthma control test [47])^A^; Self-management of asthma, (self-efficiency scale [48]^B^); In-depth aspects of asthma treatment^C^				A B C	A B C
Familial predisposition (biological parents): Allergies^A^; Diabetes^B^; Hypertension, angina pectoris, myocardial infarction, stroke^C^; Cancer^D^	A	A	A	A B C D	A B C D
Accidents requiring medical treatment^A^; Vaccine-preventable childhood diseases^B^; Infectious diseases^C^; Head and back ache^D1^; Other pain^D2^; Reproductive health^E^; Sleep duration and sleep disorders (from 18 years of age, adapted from [49])^F^; Major Health Events^G^	A B C F	A B C D1 D2 F	A B C D1 D2 F	A B D1 F G	A B D1 E F G

The questions were developed at the RKI unless stated otherwise.

**Table 4:** Contents of interviews and instruments of KiGGS Wave 2 to survey mental health

	Cross-sectional study			Cohort study	
	0-2 years	3-10 years	11-17 years	10-17 years	18-29 years
**Mental health**					
Health-related quality of life (Kidscreen-10 [50]^A^; Kidscreen-27 [50]^B^; General health survey (short form)-8, SF-8, [51, 52]^C^); Life -satisfaction (Personal Wellbeing Index Adults, PWI-A [53])^D^		A (from 7)	B	B	C D
Mental health problems in childhood and adolescence (Strengths and Differences Questionnaire, SDQ [54, 55])^A^; Diagnosed ADHD and treatment^B^; diagnoses of mental disorders in childhood and adolescence^C^; Screening for eating disorders (SCOFF Questionnaire [56])^D^		A B	A B D	A B C D	B D
Preclinical symptoms (Mental Health Inventory-5, MHI-5 + Vitality Subscale (SF-36) [51, 57])^A^; Screening for depressive disorders and panic disorders (Patient Health Questionnaire, PHQ-9 [58] and PHQ-D short version [59])^B^; Diagnoses of depression, anxiety disorders or other mental disorders in adulthood^C^					A B C
Treatment received for diagnosed mental disorders^A^; Family predisposition (biological parents): mental illness^B^				A B	A B
Self-efficacy (Scale of General Self-efficacy, SWE [60], Personal resources [61], Social support, Social Support Scale [62])^A^; Personality (age 14 and over, Big Five Inventory 10, BFI-10 [63])^B^			A	A B	A B
Adverse experiences in childhood and adolescence: Traumatisation (Childhood Trauma Questionnaire, CTQ [64])^A^; Divorce/separation/death of a parent, including dates^B^; Serious diseases/accidents in the family^C^; Mental disorders/addiction/imprisonment of member of household, experience of war/terrorism/political conflicts (adapted from [65])^D^				B C	A B C D

The questions were developed at the RKI unless stated otherwise.

**Table 5:** Contents of interviews and instruments of KiGGS Wave 2 to survey health-related behavior

	Cross-sectional study			Cohort study	
	0-2 years	3-10 years	11-17 years	10-17 years	18-29 years
**Health-related behaviour**					
Dietary habits (Food Frequency Questionnaire, FFQ [17])^A^; Breastfeeding^B^; Detailed information on early childhood nutrition^C^	B C	A B	A B	A	A
Physical and sporting activity^A^; European Health Interview Survey – Physical Activity Questionnaire, EHIS-PAQ [66-68])^B^; Daily physical activity^C^		A	A C	A	B
Smoking: tobacco use/shisha^A^; Passive smoking^B^; Use of e-cigarettes^C^; Smoking among friends^D^			A B D	A B C D	A B C D
Alcohol: harmful consumption and binge drinking (Alcohol Use Disorder Identification Test, AUDIT-C [69, 70])^A^; Alcohol abuse (Brief Alcohol Screening Instrument for Medical Care, BASIC [71])^B^			A	A	A B
Media usage^A^		A	A	A	A
Parental health-related behaviour: Sporting activity^A^; Current smoking^B^; Smoking in the presence of a child^C^; Mother’s smoking during pregnancy and breastfeeding^D^	B C D	A B C D	A B C D	A B C	

The questions were developed at the RKI unless stated otherwise.

**Table 6:** Contents of interviews and instruments of KiGGS Wave 2 to survey health care and prevention

	Cross-sectional study			Cohort study	
	0-2 years	3-10 years	11-17 years	10-17 years	18-29 years
**Health care and prevention**					
Utilisation of doctors, therapists, hospitals, ambulances ^A^; Examinations for the early detection of diseases (the so-called “U”-check-ups)^B^; Health insurance^C^	A B C	A B C	A B C	A B C	A B C
Operations^A^	A	A	A	
Regularity of brushing teeth (adapted from [72])^A^; Dental check-ups^B^; Orthodontic treatment^C^	A B	A B C	A B C	A B C	B
Health literacy (age 15 and over, Health literacy subindex [73])^A^; Early intervention (in line with [74])^B^	B		A	A	
Human papilloma virus (HPV) vaccination ♀; Reasons for non-vaccination^A^		A	♀A	♀	♀

The questions were developed at the RKI unless stated otherwise.

♀ Only for female participants

**Table 7:** Contents of interviews and instruments of KiGGS Wave 2 to survey social, family and environmental determinants

	Cross-sectional study			Cohort study	
	0-2 years	3-10 years	11-17 years	10-17 years	18-29 years
**Social, family and environmental determinants of health**					
Basic information on participants (partly adapted from [75-77]): Age and gender^A^; Current usual point of residence^B^; Usual point of residence aged 15 and born in East Germany^C^; Household composition^D^; Education^E^; Training and professional experience^F^; Occupation, household net income, employment situation^G^; Marital status, partnership^H^; Unemployment^I^; Transition periods (moving out of parental home, partnerships and family status changes, own children)^J^	A B D	A B D	A B D E	A B D E	A C D E F G H I J
Basic information on parents (adapted from [76, 77]): marital status, partnership, education, occupation, household net income, employment situation^A^	A	A	A	A	
Subjective social status [78-80]^A^			A	A	A
Family climate (Family Climate Scale [81])^A^; Adopted parenting style (Zurich brief questionnaire on parenting style, D-ZKE [82, 83])^B^; Burden on parents (adapted from [84])^C^; Well-being and personality of the parents (Personal Wellbeing Index Adults, PWI-A [53] and Big Five Inventory, BFI-10 [63])^D^; Support from outside of the family (adapted from [85])^E^; Patchwork family (past)^F^	A E	A E	A C E	A B C D F	F
Living environment (size and type of home; humidity and mould; neighbourhood/environment)^A^; Noise pollution^B^	A B	A B	A B	A B	A B
Migration background (adapted from [8, 21])^A^; Experiences of discrimination (adapted from [86])^B^	A	A	A B	A B	A B

The questions were developed at the RKI unless stated otherwise.

## References

[1] Kurth BM Ziese T Tiemann F (2005) Gesundheitsmonitoring auf Bundesebene. Bundesgesundheitsbl Gesundheitsforsch Gesundheitsschutz 48(3):261-272 edoc.rki.de/oa/articles/reu8uqsCD3T5c/PDF/28I5r4p7X3N3E.pdf (As at 25.07.2017) 10.1007/s00103-004-1001-6 15768298

[2] Kurth BM Lange C Kamtsiuris P. (2009) Gesundheitsmonitoring am Robert Koch-Institut. Sachstand und Perspektiven. Bundesgesundheitsbl Gesundheitsforsch Gesundheitsschutz edoc.rki.de/oa/articles/reAOveQSZimxU/PDF/26LMhcJK5XVZs.pdf (As at 25.07.2017) 10.1007/s00103-009-0843-3 19343279

[3] Hölling H Schlack R Kamtsiuris P. (2012) Die KiGGS-Studie. Bundesweit repräsentative Längs- und Querschnittstudie zur Gesundheit von Kindern und Jugendlichen im Rahmen des Gesundheitsmonitorings am Robert Koch-Institut. Bundesgesundheitsbl Gesundheitsforsch Gesundheitsschutz 55(6-7): 836-842 edoc.rki.de/oa/articles/relX5Hqebbmw/PDF/274v5EOZD1xJY.pdf (As at 25.07.2017) 10.1007/s00103-012-1486-3 22736165

[4] Kurth BM Kamtsiuris P Hölling H. (2016) Strategien des Robert Koch-Instituts zum Monitoring der Gesundheit von in Deutschland lebenden Kindern und Jugendlichen. Kinder- und Jugendmedizin 16(3):176-183

[5] Robert Koch-Institut und Bundeszentrale für gesundheitliche Aufklärung (Ed) (2008) Erkennen - Bewerten - Handeln: Zur Gesundheit von Kindern und Jugendlichen in Deutschland. RKI, Berlin edoc.rki.de/documents/rki_fv/relXEvoVYRBk/PDF/25VQivifMG6zQ77.pdf (As at 25.07.2017)

[6] Kurth BM Kamtsiuris P Hölling H. (2008) The challenge of comprehensively mapping children’s health in a nation-wide health survey: design of the German KiGGS-Study. BMC Public Health 8:196 18533019 10.1186/1471-2458-8-196 PMC2442072

[7] Saß AC Grüne B Brettschneider AK. (2015) Beteiligung von Menschen mit Migrationshintergrund an Gesundheitssurveys des Robert Koch-Instituts. Bundesgesundheitsbl Gesundheitsforsch Gesundheitsschutz 58(6):533-542 edoc.rki.de/oa/articles/reb2cbNSV5Xag/PDF/23Y55KHgb1WQ.pdf (As at 25.07.2017) 10.1007/s00103-015-2146-1 25896496

[8] Robert Koch-Institut (Ed) (2008) Kinder- und Jugendgesundheitssurvey (KiGGS) 2003-2006: Kinder und Jugendliche mit Migrationshintergrund in Deutschland. Beiträge zur Gesund-heitsberichterstattung des Bundes. RKI, Berlin edoc.rki.de/documents/rki_fv/reJBwqKp45PiI/PDF/23Ydv-84JGTBo6_07.pdf (As at 25.07.2017)

[9] Lange M Butschalowsky H Jentsch F. (2014) Die erste KiGGS-Folgebefragung (KiGGS Welle 1). Studiendurchführung, Stichprobendesign und Response. Bundesgesundheitsbl Gesundheitsforsch Gesundheitsschutz 57(7):747-761 edoc.rki.de/oa/articles/re5weWnRsXRSw/PDF/20B6fVTPFIdw.pdf (As at 25.07.2017) 10.1007/s00103-014-1973-9 24950824

[10] Robert Koch-Institut (Ed) (2011) KiGGS – Kinder- und Jugendgesundheitsstudie Welle 1 - Projektbeschreibung. Beiträge zur Gesundheitsberichterstattung des Bundes. RKI, Berlin edoc.rki.de/documents/rki_fv/renwMdxA9Hb7I/PDF/28RiIyKJmvRHk.pdf (As at 25.07.2017)

[11] Kamtsiuris P Lange M Rosario AS (2007) Der Kinder- und Jugendgesundheitssurvey (KiGGS): Stichprobendesign, Response und Nonresponse-Analyse. Bundesgesundheitsbl Gesundheitsforsch Gesundheitsschutz 50(5-6):547-556 edoc.rki.de/oa/articles/reeMwKaQj7lM/PDF/24z8sbCK0My3s.pdf (As at 25.07.2017) 10.1007/s00103-007-0215-9 17514438

[12] Knopf H (2007) Arzneimittelanwendung bei Kindern und Jugendlichen. Erfassung und erste Ergebnisse beim Kinder- und Jugendgesundheitssurvey (KiGGS). Bundesgesundheitsbl Gesundheitsforsch Gesundheitsschutz 50(5):863-870 edoc.rki.de/oa/articles/reEzagAaWOvY/PDF/2546Vrd7r9RC6.pdf (As at 25.07.2017) 10.1007/s00103-007-0249-z 17514472

[13] Hölling H Kamtsiuris P Lange M. (2007) Der Kinder-und Jugendgesundheitssurvey (KiGGS): Studienmanagement und Durchführung der Feldarbeit. Bundesgesundheitsbl Gesundheitsforsch Gesundheitsschutz 50(5-6):557-566 edoc.rki.de/oa/articles/rej53eEjT1Ze6/PDF/29ruDT0W37IrU.pdf (As at 25.07.2017) 10.1007/s00103-007-0216-8 17514439

[14] Schenk L Ellert U Neuhauser H (2007) Kinder und Jugendliche mit Migrationshintergrund in Deutschland. Methodische Aspekte im Kinder- und Jugendgesundheitssurvey (KiGGS). Bundesgesundheitsbl Gesundheitsforsch Gesundheitsschutz 50 (5-6):590-599 edoc.rki.de/oa/articles/reunJYxaLNDfs/PDF/233ll5mfg7L5c.pdf (As at 25.07.2017) 10.1007/s00103-007-0220-z 17514443

[15] Brauns H Scherer S Steinmann S (2003) The CASMIN Educational Classification in International Comparative Research. In: Hoffmeyer-Zlotnik JHP Wolf C (Ed) Advances in Cross-National Comparison: A European Working Book for Demographic and Socio-Economic Variables. Springer US, Boston, MA, P. 221-244

[16] Reinhardt D Petermann F (2010) Neue Morbiditäten in der Pädiatrie. Monatsschr Kinderheilkd 158(1):14-14

[17] Mensink GBM Burger M (2004) Was isst du? Ein Verzehrshäufigkeitsfragebogen für Kinder und Jugendliche. Bundesgesundheitsbl Gesundheitsforsch Gesundheitsschutz 47(3):219-226 edoc.rki.de/oa/articles/rexKPi8f0KZ3E/PDF/25okAR8peLJI.pdf (As at 25.07.2017) 10.1007/s00103-003-0794-z 15205789

[18] Kleiser C Mensink GBM Scheidt-Nave C. (2009) HuSKY: a healthy nutrition score based on food intake of children and adolescents in Germany. Br J Nutr 102(4):610-618 19203423 10.1017/S0007114509222689

[19] Lampert T Kuntz B KiGGS Study Group (2015) Growing up healthy – What significance does social status have? GBE kompakt 6(1). Robert Koch-Institut, Berlin edoc.rki.de/series/gbe-kompakt/6-1/PDF/1_en.pdf (As at 25.07.2017)

[20] Lampert T Müters S Stolzenberg H. (2014) Messung des sozioökonomischen Status in der KiGGS-Studie. Bundesgesundheitsbl Gesundheitsforsch Gesundheitsschutz 57(7):762-770 edoc.rki.de/oa/articles/reXPIrLy4LMJM/PDF/28B0RAYr9XdWs.pdf (As at 25.07.2017) 10.1007/s00103-014-1974-8 24950825

[21] Schenk L Bau AM Borde T. (2006) Mindestindikatorensatz zur Erfassung des Migrationsstatus. Bundesgesundheitsbl Gesundheitsforsch Gesundheitsschutz 49(9):853-860 edoc.rki.de/oa/articles/reiixWrKS0U/PDF/23EvY7L5ORlrE.pdf (As at 25.07.2017) 10.1007/s00103-006-0018-4 16927038

[22] Kurth BM (2012) Das RKI-Gesundheitsmonitoring – Was es enthält und wie es genutzt werden kann. Public Health Forum 20(3):4.e1-4.e3

[23] Robert Koch-Institut (Ed) (2012) Referenzperzentile für anthropometrische Maßzahlen und Blutdruck aus der Studie zur Gesundheit von Kindern und Jugendlichen in Deutschland (KiGGS). Beiträge zur Gesundheitsberichterstattung des Bundes. RKI, Berlin edoc.rki.de/documents/rki_fv/reZRsQ2J6ufLQ/PDF/28jWMa04ZjppM.pdf (As at 25.07.2017)

[24] Robert Koch-Institut (Ed) (2009) Bevölkerungsbezogene Verteilungswerte ausgewählter Laborparameter aus der Studie zur Gesundheit von Kindern und Jugendlichen in Deutschland (KiGGS). Beiträge zur Gesundheitsberichterstattung des Bundes. RKI, Berlin edoc.rki.de/documents/rki_fv/reJBwqKp45PiI/PDF/206ee9py9oog_03.pdf (As at 25.07.2017)

[25] Kahl H Dortschy R Ellsäßer G (2007) Verletzungen bei Kindern und Jugendlichen (1-17 Jahre) und Umsetzung von persönlichen Schutzmaßnahmen. Bundesgesundheitsbl Gesundheitsforsch Gesundheitsschutz 50(5):718-727 edoc.rki.de/oa/articles/re5D5gfxZr3AY/PDF/27CbOnjqoeIQ.pdf (As at 25.07.2017) 10.1007/s00103-007-0233-7 17514456

[26] Schlaud M Atzpodien K Thierfelder W (2007) Allergische Erkrankungen. Ergebnisse aus dem Kinder- und Jugendgesundheitssurvey (KiGGS). Bundesgesundheitsbl Gesundheitsforsch Gesundheitsschutz 50(5-6):701-710 edoc.rki.de/oa/articles/reRhnrND9xOGA/PDF/27Kp0kRUqJI.pdf (As at 28.07.2017) 10.1007/s00103-007-0231-9 17514454

[27] Hölling H Erhart M Ravens-Sieberer U. (2007) Verhaltensauffälligkeiten bei Kindern und Jugendlichen. Erste Ergebnisse aus dem Kinder- und Jugendgesundheitssurvey (KiGGS). Bundesgesundheitsbl Gesundheitsforsch Gesundheitsschutz 50(5/6):784-793 edoc.rki.de/oa/articles/reryPJPcmUGw/PDF/25maWiJoxrkYE.pdf (As at 28.07.2017) 10.1007/s00103-007-0241-7 17514464

[28] Schlack R Hölling H Kurth BM. (2007) Die Prävalenz der Aufmerksamkeitsdefizit-/Hyperaktivitätsstörung (ADHS) bei Kindern und Jugendlichen in Deutschland. Erste Ergebnisse aus dem Kinder- und Jugendgesundheitssurvey (KiGGS). Bundesgesundheitsbl Gesundheitsforsch Gesundheitsschutz 50(5/6):827-835 edoc.rki.de/oa/articles/reuPv4KL2czE/PDF/227Ar6DRSOXo.pdf (As at 28.07.2017) 10.1007/s00103-007-0246-2 17514469

[29] Kurth BM Schaffrath Rosario A (2007) Die Verbreitung von Übergewicht und Adipositas bei Kindern und Jugendlichen in Deutschland. Ergebnisse des bundesweiten Kinder- und Jugendgesundheitssurveys (KiGGS). Bundesgesundheitsbl Gesundheitsforsch Gesundheitsschutz 50(5-6):736-743 edoc.rki.de/oa/articles/reryPJPcmUGw/PDF/20pyWvIPNYV52.pdf (As at 28.07.2017) 10.1007/s00103-007-0235-5 17514458

[30] Lampert T (2011) Soziale Ungleichheit und Gesundheit im Kindes- und Jugendalter. Pädiatrie up2date 6(02):119-142

[31] Robert Koch-Institut (Ed) (2010) Gesundheitliche Ungleichheit bei Kindern und Jugendlichen in Deutschland. Beiträge zur Gesundheitsberichterstattung des Bundes. RKI, Berlin edoc.rki.de/documents/rki_fv/reQXTR7OSGFRg/PDF/29lllSiUWs.pdf (As at 25.07.2017)

[32] Robert Koch-Institut (Ed) (2017) Gesundheitliche Ungleichheit in verschiedenen Lebensphasen. Gesundheitsberichterstattung des Bundes. Gemeinsam getragen von RKI und Destatis. RKI, Berlin edoc.rki.de/documents/rki_fv/releGa5LqOxGE/PDF/25xIYiGiDQ6x2w.pdf (As at 28.07.2017)

[33] Schmitz R Thamm M Ellert U. (2014) Verbreitung häufiger Allergien bei Kindern und Jugendlichen in Deutschland: Ergebnisse der KiGGS-Studie - Erste Folgebefragung (KiGGS Welle 1). Bundesgesundheitsbl Gesundheitsforsch Gesundheitsschutz 57(7):771-778 edoc.rki.de/oa/articles/reanlTxmpPiBk/PDF/27CDfhKBFstMs.pdf (As at 28.07.2017) 10.1007/s00103-014-1975-7 24950826

[34] Brettschneider AK Schienkiewitz A Schmidt S. (2017) Updated prevalence rates of overweight and obesity in 4- to 10-year-old children in Germany. Results from the telephone-based KiGGS Wave 1 after correction for bias in parental reports. Eur J Pediatr 176(4):547-551 28132095 10.1007/s00431-017-2861-8

[35] Brettschneider AK Schaffrath Rosario A Kuhnert R. (2015) Updated prevalence rates of overweight and obesity in 11- to 17-year-old adolescents in Germany. Results from the telephone-based KiGGS Wave 1 after correction for bias in selfreports. BMC Public Health 15(1):1-9 26541820 10.1186/s12889-015-2467-x PMC4636076

[36] Hölling H Schlack R Petermann F. (2014) Psychische Auffälligkeiten und psychosoziale Beeinträchtigungen bei Kindern und Jugendlichen im Alter von 3 bis 17 Jahren in Deutschland – Prävalenz und zeitliche Trends zu 2 Erhebungszeitpunkten (2003-2006 und 2009-2012). Bundesgesundheitsbl Gesundheitsforsch Gesundheitsschutz 57(7):807-819 10.1007/s00103-014-1979-3 24950830

[37] Poethko-Müller C Schmitz R Ellert U (2015) Die aktuelle Situation der Gesundheit von Kindern und Jugendlichen. Public Health Forum 23(1):30

[38] Rattay P Starker A Domanska O. (2014) Trends in der Inanspruchnahme ambulant-ärztlicher Leistungen im Kindesund Jugendalter: Ergebnisse der KiGGS-Studie – Ein Vergleich von Basiserhebung und erster Folgebefragung (KiGGS Welle 1). Bundesgesundheitsbl Gesundheitsforsch Gesundheitsschutz 57(7):878-891 10.1007/s00103-014-1989-1 24950837

[39] Lampert T Kuntz B KiGGS Study Group (2014) Tabak- und Alkoholkonsum bei 11- bis 17-jährigen Jugendlichen: Ergebnisse der KiGGS-Studie - Erste Folgebefragung (KiGGS Welle 1). Bundesgesundheitsbl Gesundheitsforsch Gesundheitsschutz 57(7):830-839 edoc.rki.de/oa/articles/reAq3DgSjnNxU/PDF/23aKgb9SIyu2.pdf (As at 28.07.2017) 10.1007/s00103-014-1982-8 24950832

[40] Kuntz B Lampert T (2016) Social disparities in parental smoking and young children’s exposure to secondhand smoke at home: a time-trend analysis of repeated cross-sectional data from the German KiGGS study between 2003-2006 and 2009-2012. BMC Public Health 16:485 27277721 10.1186/s12889-016-3175-x PMC4898452

[41] Kuntz B Lampert T (2016) Social Disparities in Maternal Smoking during Pregnancy: Comparison of Two Birth Cohorts (1996-2002 and 2003-2012) Based on Data from the German KiGGS Study. Geburtshilfe Frauenheilkd 76(3):239-247 27065485 10.1055/s-0042-100207 PMC4824631

[42] Bundesministerium für Ernährung Landwirtschaft und Verbraucherschutz, Bundesministerium für Gesundheit (2008) IN FORM. Deutschlands Initiative für gesunde Ernährung und mehr Bewegung. BMELV, BMG, Berlin

[43] Bundesministerium für Gesundheit (Ed) (2010) Nationales Gesundheitsziel „Gesund aufwachsen: Lebenskompetenz, Bewegung, Ernährung“. BMG, Berlin

[44] Bundesministerium für Gesundheit (Ed) (2017) Nationales Gesundheitsziel „Rund um die Geburt“. BMG, Berlin

[45] Cox B van Oyen H Cambois E. (2009) The reliability of the Minimum European Health Module. Int J Public Health 54(2):55-60 19183846 10.1007/s00038-009-7104-y

[46] Bethell CD Read D Stein RE. (2002) Identifying children with special health care needs: development and evaluation of a short screening instrument. Ambul Pediatr 2(1):38-48 11888437 10.1367/1539-4409(2002)002<0038:icwshc>2.0.co;2

[47] Nathan RA Sorkness CA Kosinski M. Development of the asthma control test. J Allergy Clin Immunol 113(1):59-65 14713908 10.1016/j.jaci.2003.09.008

[48] Freund T Gensichen J Goetz K. (2013) Evaluating self-efficacy for managing chronic disease: psychometric properties of the six-item Self-Efficacy Scale in Germany. J Eval Clin Pract 19(1):39-43 21883720 10.1111/j.1365-2753.2011.01764.x

[49] Riemann D Backhaus J (1996) Behandlung von Schlafstörungen. Beltz Psychologie Verlags Union, Weinheim

[50] The KIDSCRREN Group Europe (Ed) (2006) The KIDSCREEN Questionnaires: Quality of life questionnaires for children and adolescents. Handbook. Pabst Science Publishers, Lengerich

[51] Ware JE Kosinski M Bjorner JB. (2007) User’s manual for the SF-36v2 health survey, 2nd edition. Quality Metric Incorporated, Lincoln, RI

[52] Ellert U Lampert T Ravens-Sieberer U (2005) Measuring health-related quality of life with the SF-8. Bundesgesundheitsbl Gesundheitsforsch Gesundheitsschutz 48(12):1330-1337 10.1007/s00103-005-1168-5 16270186

[53] International Wellbeing Group (2006) Personal Wellbeing Index. Australian Centre on Quality of Life, Deakin University, Melbourne

[54] Goodman R (1997) The Strengths and Difficulties Questionnaire: a research note. J Child Psychol Psychiatry 38(5):581-586 9255702 10.1111/j.1469-7610.1997.tb01545.x

[55] Goodman R (1999) The extended version of the Strengths and Difficulties Questionnaire as a guide to child psychiatric caseness and consequent burden. J Child Psychol Psychiatry 40(5):791-799 10433412

[56] Morgan JF Reid F Lacey JH (1999) The SCOFF questionnaire: assessment of a new screening tool for eating disorders. BMJ 319(7223):1467-1468 10582927 10.1136/bmj.319.7223.1467 PMC28290

[57] Rumpf HJ Meyer C Hapke U. (2001) Screening for mental health: validity of the MHI-5 using DSM-IV Axis I psychiatric disorders as gold standard. Psychiatry research 105(3):243-253 11814543 10.1016/s0165-1781(01)00329-8

[58] Kroenke K Spitzer R.L. (2002) The PHQ-9: A New Depression Diagnostic an Severity Measure. Psychiatr Ann 32(9):509-515

[59] Gräfe K Zipfel S Herzog W. (2004) Screening psychischer Störungen mit dem „Gesundheitsfragebogen für Patienten (PHQ-D)“. Diagnostica 50(4):171-181

[60] Schwarzer R (2003) SWE - Skala zur Allgemeinen Selbstwirksamkeitserwartung. In: Brähler E Schumacher J Strauß B (Ed) Diagnostische Verfahren in der Psychotherapie. Hogrefe, Göttingen

[61] Bettge S Ravens-Sieberer U (2003) Schutzfaktoren für die psychische Gesundheit von Kindern und Jugendlichen – empirische Ergebnisse zur Validierung eines Konzepts. Gesundheitswesen 65(03):167-172 12698386 10.1055/s-2003-38514

[62] Dalgard OS Tambs K (1995) Social support, negative life events and mental health. Br J Psychiatry 166(1):29-34 7894872 10.1192/bjp.166.1.29

[63] Rammstedt B Kemper CJ Klein MC. (2013) Eine kurze Skala zur Messung der fünf Dimensionen der Persönlichkeit. Methoden, Daten, Analysen (mda) 7(2): 233-249

[64] Klinitzke G Romppel M Häuser W. (2012) Die deutsche Version des Childhood Trauma Questionnaire (CTQ) – psychometrische Eigenschaften in einer bevölkerungsrepräsentativen Stichprobe. Psychother Psychosom Med Psychol 62(2):47-51 22203470 10.1055/s-0031-1295495

[65] World Health Organization (2014) Adverse Childhood Experiences International Questionnaire (ACE-IQ). WHO international: Violence and Injury Prevention www.who.int/violence_injury_prevention/violence/activities/adverse_childhood_experiences/questionnaire.pdf?ua=1 (As at 30.06.2017)

[66] Baumeister SE Ricci C Kohler S. (2016) Physical activity surveillance in the European Union: reliability and validity of the European Health Interview Survey-Physical Activity Questionnaire (EHIS-PAQ). Int J Behav Nutr Phys Act 13(1):61 27215626 10.1186/s12966-016-0386-6 PMC4877949

[67] Finger JD Gisle L Mimilidis H. (2015) How well do physical activity questions perform? A European cognitive testing study. Arch Public Health 73(1):57 26629340 10.1186/s13690-015-0109-5 PMC4665945

[68] Finger JD Tafforeau J Gisle L. (2015) Development of the European Health Interview Survey - Physical Activity Questionnaire (EHIS-PAQ) to monitor physical activity in the European Union. Arch Public Health 73(1):59 26634120 10.1186/s13690-015-0110-z PMC4667448

[69] Bradley KA McDonell MB Bush K. (1998) The AUDIT alcohol consumption questions: reliability, validity, and responsiveness to change in older male primary care patients. Alcohol Clin Exp Res 22(8):1842-1849 9835306 10.1111/j.1530-0277.1998.tb03991.x

[70] Bush K Kivlahan DR McDonell MB. (1998) The AUDIT alcohol consumption questions (AUDIT-C): an effective brief screening test for problem drinking. Ambulatory Care Quality Improvement Project (ACQUIP). Alcohol Use Disorders Identification Test. Arch Intern Med 158(16):1789-1795 9738608 10.1001/archinte.158.16.1789

[71] Bischof G Reinhardt S Grothues J. (2007) Development and Evaluation of a Screening Instrument for Alcohol-Use Disorders and At-Risk Drinking: The Brief Alcohol Screening Instrument for Medical Care (BASIC). J Stud Alcohol Drugs 68(4):607-614 17568967 10.15288/jsad.2007.68.607

[72] Micheelis W Schiffner U (2006) Vierte Deutsche Mundgesundheitsstudie (DMS IV). Institut der Deutschen Zahnärzte IDZ, Materialreihe Band 31. Deutscher Ärzte-Verlag, Köln

[73] Röthlin F Pelikan JM G K (2013) Die Gesundheitskompetenz der 15-jährigen Jugendlichen in Österreich. Abschlussbericht der österreichischen Gesundheitskompetenz Jugendstudie im Auftrag des Hauptverbands der österreichischen Sozialversicherungsträger (HVSV). Ludwig Boltzmann Institut Health Promotion Research (LBIHPR), Wien

[74] Lang K Brand C Renner I. (2015) Wie werden Angebote der Frühen Hilfen genutzt? Erste Daten aus den Pilotstudien der Prävalenz- und Versorgungsstudie. In: Nationales Zentrum Frühe Hilfen (NZFH), Forschungsverbund Deutsches Jugendinstitut e.V. (DJI), Dortmund TU. (Ed) Datenreport Frühe Hilfen. Nationales Zentrum Frühe Hilfen, Köln

[75] Statistical Office of the European Union (Eurostat) (2016) Income and living conditions (EU-SILC) ec.europa.eu/eurostat/web/income-and-living-conditions/overview (As at 16.6.2017)

[76] Lampert T Kroll LE (2009) Die Messung des sozioökonomischen Status in sozialepidemiologischen Studien. In: Richter M Hurrelmann K (Ed) Gesundheitliche Ungleichheit – Grundlagen, Probleme, Perspektiven, 2. aktualisierte Auflage. VS Verlag für Sozialwissenschaften, Wiesbaden, P. 309-334

[77] Statistisches Bundesamt (Ed) (2016) Demographische Stan dards. Ausgabe 2016 Statistik und Wissenschaft, Bd 17. Destatis, Wiesbaden

[78] Goodman E Adler NE Kawachi I. (2001) Adolescents Perceptions of Social Status: Development and Evaluation of a New Indicator. Pediatrics 108(2):e31 11483841 10.1542/peds.108.2.e31

[79] Adler NE Epel ES Castellazzo G. (2000) Relationship of subjective and objective social status with psychological and physiological functioning: preliminary data in healthy white women. Health Psychol 19(6):586-592 11129362 10.1037//0278-6133.19.6.586

[80] Hoebel J Müters S Kuntz B. (2015) Messung des subjektiven sozialen Status in der Gesundheitsforschung mit einer deutschen Version der MacArthur Scale. Bundesgesundheitsbl Gesundheitsforsch Gesundheitsschutz 58(7):749-757 10.1007/s00103-015-2166-x 25986532

[81] Schneewind K Beckmann M Hecht-Jackl A (1985) Das FK-Testsystem. Das Familienklima aus der Sichtweise der Eltern und der Kinder. Forschungsberichte aus dem Institutsbereich Persönlichkeitspsychologie und Psychodiagnostik, Nr. 8.1. Ludwig-Maximilians-Universität, München

[82] Reitzle M (2015) D-ZKE (vormals ZKE). Zürcher Kurzfragebogen zum Erziehungsverhalten (deutsche Neunormierung). In: Richter D Brähler E Ernst J (Ed) Diagnostische Verfahren für Beratung und Therapie von Paaren und Familien. Hogreve, Göttingen

[83] Reitzle M Winkler-Metzke C Steinhausen HC (2001) Eltern und Kinder: Der Zürcher Kurzfragebogen zum Erziehungsverhalten (ZKE). Diagnostica 47(4):196-207

[84] Sperlich S Arnhold-Kerri S Geyer S (2011) Soziale Lebenssituation und Gesundheit von Müttern in Deutschland. Ergebnisse einer Bevölkerungsstudie. Bundesgesundheitsbl Gesundheitsforsch Gesundheitsschutz 54(6):735-744 10.1007/s00103-011-1283-4 21626379

[85] Tietze W Lee H-J Bensel J. (in Vorb.) Pädagogische Qualität in Kindertageseinrichtungen und- Kindertagespflegestellen. In: Tietze W (Ed) NUBBEK – Nationale Untersuchung zur Bildung, Betreuung und Erziehung in der frühen Kindheit

[86] Deutsches Jugendinstitut (DJI) München, Technische Universität Chemnitz-Zwickau (2001) DJI-Ausländersurvey – Jugendliche (10/1996-02/1997). GESIS Datenarchiv dbk.gesis.org/dbksearch/SDESC2.asp?no=3371&search=3371&search2=&DB=D (As at 28.07.2017)

